# 5-HT receptors exert differential effects on seizure-induced respiratory arrest in DBA/1 mice

**DOI:** 10.1371/journal.pone.0304601

**Published:** 2024-05-31

**Authors:** Yundan Pan, Zheren Tan, Jialing Guo, Hua-Jun Feng

**Affiliations:** 1 Department of Anesthesiology, Xiangya Hospital, Central South University, Changsha, China; 2 Department of Anesthesia, Critical Care and Pain Medicine, Massachusetts General Hospital and Harvard Medical School, Boston, MA, United States of America; 3 Department of Critical Care Medicine, Xiangya Hospital, Central South University, Changsha, China; 4 National Health Commission Key Laboratory of Birth Defect Research and Prevention, Hunan Provincial Maternal and Child Health Care Hospital, Changsha, China; University of Modena and Reggio Emilia, ITALY

## Abstract

Both clinical and animal studies demonstrated that seizure-induced respiratory arrest (S-IRA) contributes importantly to sudden unexpected death in epilepsy (SUDEP). It has been shown that enhancing serotonin (5-HT) function relieves S-IRA in animal models of SUDEP, including DBA/1 mice. Direct activation of 5-HT_3_ and 5-HT_4_ receptors suppresses S-IRA in DBA/1 mice, indicating that these receptors are involved in S-IRA. However, it remains unknown if other subtypes of 5-HT receptors are implicated in S-IRA in DBA/1 mice. In this study, we investigated the action of an agonist of the 5-HT_1A_ (8-OH-DPAT), 5-HT_2A_ (TCB-2), 5-HT_2B_ (BW723C86), 5-HT_2C_ (MK-212), 5-HT_6_ (WAY-208466) and 5-HT_7_ (LP-211) receptor on S-IRA in DBA/1 mice. An agonist of the 5-HT receptor or a vehicle was intraperitoneally administered 30 min prior to acoustic simulation, and the effect of each drug/vehicle on the incidence of S-IRA was videotaped for offline analysis. We found that the incidence of S-IRA was significantly reduced by TCB-2 at 10 mg/kg (30%, n = 10; *p* < 0.01, Fisher’s exact test) but was not altered by other agonists compared with the corresponding vehicle controls in DBA/1 mice. Our data demonstrate that 5-HT_2A_ receptors are implicated in S-IRA, and 5-HT_1A_, 5-HT_2B_, 5-HT_2C_, 5-HT_6_ and 5-HT_7_ receptors are not involved in S-IRA in DBA/1 mice.

## Introduction

Sudden unexpected death in epilepsy (SUDEP) is the most severe complication of epilepsy that ranks second among neurological disorders in years of potential life lost [1]. SUDEP mainly occurs in epilepsy patients at younger ages, in which the risk of sudden death is 24- to 28-fold higher than that in the general population [1,2]. Although several pathophysiological mechanisms are proposed to contribute to the SUDEP [3–10], cardiorespiratory and arousal deficits have received large attention [11,12]. Both clinical and animal studies demonstrated that seizure-induced respiratory arrest (S-IRA) contributes importantly to death in most SUDEP cases [13–26].

Previous studies showed that serotonergic (5-HT) neurotransmission plays an important role in the pathogenesis of the S-IRA in provoked seizure models, including the DBA/1 mouse [6,20,21,27,28]. There are fourteen 5-HT receptor subtypes identified up to date [29], among which the expression of 5-HT_2B_, 5-HT_2C_, 5-HT_3_ and 5-HT_4_ receptors is altered in DBA/1 and DBA/2 mice [28,30]. Consistent with this, systemic administration of SR 57227, a 5-HT_3_ agonist, suppresses S-IRA, and the S-IRA-suppressing effect of fluoxetine, a selective 5-HT reuptake inhibitor, is prevented by a 5-HT_3_ antagonist, ondansetron in DBA/1 mice [31]. It was also reported that the action of fenfluramine, a 5-HT releaser and agonist [32], to prevent S-IRA in DBA/1 mice is primarily mediated by the activation of 5-HT_4_ receptors, and directly activating 5-HT_4_ receptors by its agonist BIMU-8 lowers the incidence of S-IRA [33]. These preceding studies suggest that 5-HT_3_ and 5-HT_4_ receptors are involved in S-IRA in DBA/1 mice. However, it remains unknown whether other 5-HT receptor subtypes contribute to S-IRA in DBA/1 mice.

In the current study, we tested the effect of activating 5-HT_1A_, 5-HT_2A_, 5-HT_2B_, 5-HT_2C_, 5-HT_6_ or 5-HT_7_ receptors on S-IRA in DBA/1 mice, as these 5-HT receptors were shown to modulate breathing and arousal [34–37] or altered in these mice [28].

## Materials and methods

### Animals

DBA/1 mice purchased initially from Envigo (Indianapolis, IN, USA), were maintained and bred in the Massachusetts General Hospital animal facility, with *ad libitum* access to standard rodent food and water. The animal facility is temperature- and humidity-controlled with a 12-h light/dark cycle. The care and use of animals strictly adhered to the National Institutes of Health Guide for the Care and Use of Laboratory Animals. As this study aimed to investigate the role of 5-HT receptors in seizure-induced sudden death in DBA/1 mice, death was used as an endpoint, and all experimental procedures, including death as an endpoint, were approved by the Institutional Animal Care and Use Committee (IACUC) of Massachusetts General Hospital (animal protocol # 2012N000024). The health and behavior of the DBA/1 mice were monitored once daily. If a DBA/1 mouse exhibited an inability to ambulate/maintain an upright position, uncontrolled bleeding, prolonged inappetence/marked dehydration or inability to eat/drink, it was immediately euthanized. No DBA/1 mice died before meeting these criteria for euthanasia. DBA/1 mice were gently handled, and every effort ensured minimal pain and discomfort throughout the experiments. All animal welfare considerations were taken, and every effort was made to minimize the number of mice used in the study.

### Seizure-induced respiratory arrest (S-IRA)

Generalized audiogenic seizures and S-IRA were evoked by acoustic stimulation using an electrical bell (96 dB SPL) in DBA/1 mice, as previously described [38]. Briefly, a DBA/1 mouse was primed by daily exposure to acoustic stimulation for 3–4 days starting from postnatal day 26. Once a DBA/1 mouse exhibited S-IRA after generalized audiogenic seizures and was resuscitated using a rodent ventilator, it became susceptible to S-IRA in subsequent tests. Primed DBA/1 mice of both sexes at ∼two months of age were used in the experiments. All DBA/1 mice tested were included in the analysis. After completion of the investigation, the surviving DBA/1 mice were euthanized using carbon dioxide.

### Drug treatments

8-OH-DPAT (5-HT_1A_ receptor agonist), BW723C86 (5-HT_2B_ agonist), WAY-208466 dihydrochloride (5-HT_6_ agonist) and LP-211 (5-HT_7_ agonist) were purchased from Millipore Sigma (St. Louis, MO, USA). TCB-2 (5-HT_2A_ agonist) and MK-212 hydrochloride (5-HT_2C_ agonist) were purchased from Tocris (Minneapolis, MN, USA). BW723C86 was dissolved in 5% tween 80 and 95% saline and LP-211 was dissolved in 2% dimethyl sulfoxide (DMSO) and 98% saline. All other 5-HT receptor agonists were dissolved in saline.

Twenty-four hours before a drug or vehicle treatment, the susceptibility of a primed DBA/1 mouse to S-IRA was confirmed by acoustic stimulation. If the DBA/1 mouse displayed S-IRA, a 5-HT receptor agonist or vehicle was intraperitoneally (i.p.) administered 30 min prior to acoustic stimulation. The effect of the 5-HT receptor agonist or the vehicle on the incidence of S-IRA and seizure behaviors in primed DBA/1 mice were videotaped for offline analysis.

### Statistical analysis

Data were analyzed using Prism 5.0f software (GraphPad Software Inc., La Jolla, CA, USA). The incidence of S-IRA between the drug treatment and vehicle control groups or between the male and female DBA/1 mice was compared using Fisher’s exact test. *p* < 0.05 was considered indicative of a significant difference.

## Results

### Effects of 5-HT_2A_, 5-HT_2B_ and 5-HT_2C_ receptor agonists on S-IRA

**Fig 1:**
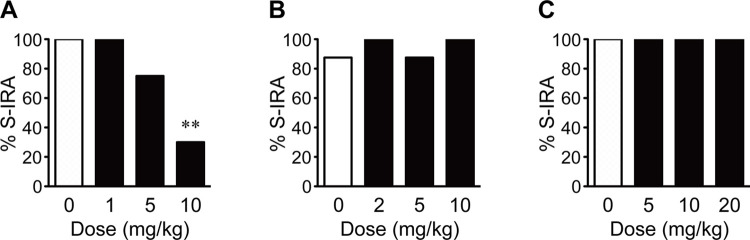
Activating 5-HT_2A_ but not 5-HT_2B_ and 5-HT_2C_ receptors suppresses S-IRA in DBA/1 mice. A) Systemic administration of the 5-HT_2A_ receptor agonist TCB-2 significantly reduced the incidence of S-IRA at 10 mg/kg compared with the vehicle control (dose zero). But, TCB-2 at 5 mg/kg or 1 mg/kg was ineffective in alleviating S-IRA. B, C) Injection of the 5-HT_2B_ receptor agonist BW723C86 at 2–10 mg/kg or 5-HT_2C_ receptor agonist MK-212 at 5–20 mg/kg exerted no effect on S-IRA compared with the corresponding vehicle control, respectively. ** *p* < 0.01: Significantly different from the vehicle control (dose zero) (Fisher’s exact test).

As compared with the incidence of S-IRA in the vehicle control group (100%, n = 9), i.p. administration of TCB-2, a 5-HT_2A_ receptor agonist, significantly reduced that 30 min after injection at 10 mg/kg (30%, n = 10) (*p* < 0.01) in DBA/1 mice. No significant sex-dependent effect of TCB-2 on S-IRA was observed. The incidence of S-IRA was not significantly suppressed by TCB-2 at 5 mg/kg (75%, n = 8) and 1 mg/kg (100%, n = 8) compared with the vehicle control (Fig 1A). However, i.p. injection of BW723C86, a 5-HT_2B_ agonist, at 10 mg/kg (100%, n = 8), 5 mg/kg (87.5%, n = 8) and 2 mg/kg (100%, n = 8) did not significantly alleviate S-IRA compared with the vehicle control (87.5%, n = 8) in DBA/1 mice (Fig 1B). Compared with the vehicle control (100%, n = 9), administration of MK-212, a 5-HT_2C_ agonist, at 20 mg/kg (100%, n = 9), 10 mg/kg (100%, n = 8) and 5 mg/kg (100%, n = 8) did not significantly modify S-IRA in DBA/1 mice (Fig 1C).

### Effects of 5-HT_1A_, 5-HT_6_ and 5-HT_7_ receptor agonists on S-IRA

**Fig 2:**
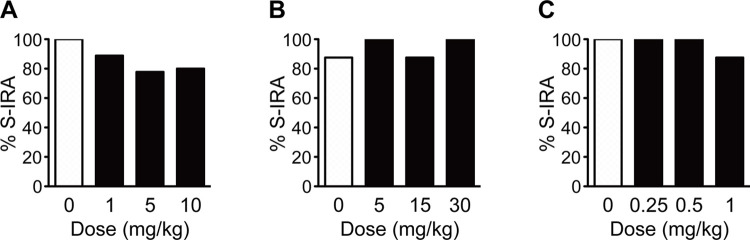
Stimulating the function of 5-HT_1A_, 5-HT_6_ and 5-HT_7_ receptors engenders no effect on S-IRA in DBA/1 mice. A, B, C) Systemic injection of the 5-HT_1A_ receptor agonist 8-OH-DPAT at 1–10 mg/kg, 5-HT_6_ receptor agonist WAY-208466 at 5–30 mg/kg and 5-HT_7_ receptor agonist LP-211 at 0.25–1 mg/kg did not significantly alter the incidence of S-IRA compared with the corresponding vehicle control (dose zero) in DBA/1 mice, respectively. (Fisher’s exact test).

Administration of 8-OH-DPAT, a 5-HT_1A_ agonist, at 10 mg/kg (80%, n = 10), 5 mg/kg (77.8%, n = 9) and 1 mg/kg (88.9%, n = 9) did not significantly relieve S-IRA compared with the vehicle control (100%, n = 9) in DBA/1 mice (Fig 2A). Compared with the vehicle control (87.5%, n = 8), WAY-208466, a 5-HT_6_ agonist, at 30 mg/kg (100%, n = 8), 15 mg/kg (87.5%, n = 8) and 5 mg/kg (100%, n = 8) did not significantly alter S-IRA in DBA/1 mice (Fig 2B). Injection of LP-211, a 5-HT_7_ agonist, at 1 mg/kg (87.5%, n = 8), 0.5 mg/kg (100%, n = 8) and 0.25 mg/kg (100%, n = 8) did not significantly change S-IRA compared with the vehicle control (100%, n = 8) in DBA/1 mice (Fig 2C).

## Discussion

In the present study, we demonstrated that the activation of 5-HT_2A_ receptors suppresses S-IRA in DBA/1 mice. However, selective stimulation of 5-HT_1A_, 5-HT_2B_, 5-HT_2C_, 5-HT_6_ or 5-HT_7_ receptors produces no effect on S-IRA. These data suggest that 5-HT_2A_ receptors are implicated in S-IRA, but 5-HT_1A_, 5-HT_2B_, 5-HT_2C_, 5-HT_6_ and 5-HT_7_ receptors are not involved in S-IRA in DBA/1 mice. The present findings add to the previous studies on 5-HT_2_ agonists [20] with the addition of evaluating several other 5-HT receptor agonists and demonstrate the generality of the results across different SUDEP models, which is useful because none of the models perfectly mimics human SUDEP.

Clinical studies observed that SUDEP patients exhibit breathing difficulty after generalized tonic-clonic seizures [13–17], followed by respiratory arrest and subsequent asystole [19]. In line with this, S-IRA occurs before cardiac arrhythmia and asystole in several provoked and spontaneous seizure models [18,20–23,25]. In addition, S-IRA can be resuscitated using a rodent ventilator in the DBA/1 mice [5]. These preceding studies show that S-IRA is the primary cause of death in most SUDEP cases. Enhancing 5-HT neurotransmission by administering selective 5-HT reuptake inhibitors [20,27,39,40], augmenting 5-HT synthesis [21] or stimulating 5-HT neurons in the dorsal raphe [6] prevents S-IRA. Clinical studies also indicate that elevated 5-HT levels potentially protect against SUDEP in patients with epilepsy [41,42]. Although 5-HT receptors regulate breathing [34], our current data do not support the idea that elevated 5-HT neurotransmission prevents S-IRA by directly stimulating the medullary respiratory center to enhance breathing. Among the 5-HT receptors tested in this study, 5-HT_1A_, 5-HT_2A_, 5-HT_2B_ and 5-HT_7_ receptors are expressed in the medullary respiratory center [34–36]. In the current study, we observed that stimulating the function of 5-HT_2A_ receptors relieves S-IRA in DBA/1 mice, consistent with a previous study using an electroshock model [20]. Another study also found that the reduction of S-IRA evoked by selective stimulation of 5-HT signaling was blocked by a 5-HT_2_ antagonist in DBA/1 mice [43]. If the S-IRA-suppressing effect of the 5-HT_2A_ agonist TCB-2 were mediated by its action in the medullary respiratory center to augment breathing, we would expect that activation of 5-HT_1A_, 5-HT_2B_ or 5-HT_7_ receptors also reduced S-IRA in DBA/1 mice, as stimulation of these receptors was shown to promote breathing [34,35]. However, activating 5-HT_1A_ and 5-HT_2B_ receptors in the current study or stimulating 5-HT_7_ receptors in the current study and previous research using a different agonist [44] engenders no effect on S-IRA in DBA/1 mice. This finding echoes our earlier observation that fluoxetine at a dose effective in reducing S-IRA produces no effect on basal breathing in anesthetized or behaving DBA/1 mice and that potent breathing stimulants exert no protective effects on S-IRA in DBA/1 mice [40]. This finding also aligns with a previous neuroimaging study showing that enhanced 5-HT signaling by fluoxetine, a selective 5-HT reuptake inhibitor, does not alter the activity of the medullary respiratory center [45].

Previous studies revealed that most patients with SUDEP die in the prone position [46–48], indicating that a deficit in arousal response may contribute to SUDEP. The activation of 5-HT_2A_ receptors induces arousal [49], consistent with the current study that the 5-HT_2A_ receptor agonist reduces S-IRA in DBA/1 mice. The brainstem dorsal raphe mediates the arousal response [50]. Our study found that selective stimulation of 5-HT neurons in the dorsal raphe using optogenetics suppresses S-IRA in DBA/1 mice, mediated by 5-HT_3_ receptors [6]. These studies suggest that the arousal response or the arousal-associated mechanism is involved in the pathogenesis of S-IRA. In accord with this, it was reported that the activity of dorsal raphe during hypercapnic challenges is elevated in patients with epilepsy compared with healthy controls [51]. Furthermore, this idea is supported by our observation that enhancing the function of norepinephrine, another monoamine involved in arousal [52], alleviates S-IRA via activating α2 adrenoceptors in DBA/1 mice [38,53,54]. That said, stimulating the function of 5-HT_6_ receptors exerts no effect on S-IRA in DBA/1 mice in the current study, although 5-HT_6_ receptor activation was reported to enhance arousal [37]. However, it was reported that the effect of 5-HT_6_ receptor activation differs from other 5-HT receptors, suggesting that each 5-HT receptor subtype makes a specific contribution to the forebrain activity [55]. The null mutant mice lacking 5-HT_2C_ receptors are susceptible to audiogenic seizures [56], and 5-HT_2C_ knockout mice exhibit spontaneous seizures with late onset of SUDEP [57]. Also, the expression of 5-HT_2C_ receptors is reduced in DBA/1 mice [28]. These studies suggest that 5-HT_2C_ receptors may be involved in S-IRA. Surprisingly, our current research and a previous report [20] show that activation of 5-HT_2C_ receptors engenders no effect on S-IRA. This observation indicates that the pathophysiological mechanisms that may not be associated with 5-HT signaling also contribute to S-IRA.

The strength of this study is to use the selective agonist of several 5-HT receptor subtypes to determine the 5-HT receptors involved in S-IRA. Also, the differential effect of activating 5-HT receptors on S-IRA helps map the brain structures that potentially contribute to the protective action of enhanced 5-HT signaling on S-IRA. The limitations of this study include that the DBA/1 mouse is a provoked seizure model, which does not closely mimic SUDEP in humans that results from spontaneous seizures, and that the specific action of these 5-HT agonists in the respiratory brainstem network was not investigated. Further studies are needed to confirm these findings in SUDEP models with spontaneous seizures and explore where these 5-HT agonists act in the respiratory network.

## Conclusions

This study demonstrates that a selective 5-HT_2A_ receptor agonist reduces S-IRA, but an agonist of the 5-HT_1A_, 5-HT_2B_, 5-HT_2C_, 5-HT_6_ or 5-HT_7_ receptor exerts no effect on S-IRA in DBA/1 mice. These data indicate that 5-HT_2A_ receptors appear to play an important role in the control of S-IRA and have the potential to be useful in the control of SUDEP. This finding contrasts with the lack of effects in 5-HT_1A_, 5-HT_2B_, 5-HT_2C_, 5-HT_6_ and 5-HT_7_ receptors, which are not likely to be useful.

## Supporting information

S1 File Datasets for calculating the incidence of seizure-induced respiratory arrest (S-IRA) for each 5-HT agonist in DBA/1 mice. (XLSX)
